# ASSOCIATIONS BETWEEN DISCRIMINATION AND COGNITION AMONG INDIGENOUS, BLACK, HISPANIC, AND WHITE OLDER ADULTS

**DOI:** 10.1093/geroni/igae098.0895

**Published:** 2024-12-31

**Authors:** Cliff Whetung, Ernest Gonzales

**Affiliations:** University of Minnesota, Duluth, Duluth, Minnesota, United States; New York University, New York, United States

## Abstract

The number of Indigenous older adults will more than double in the next thirty years, and they are at high risk of cognitive pathologies in later life. This study centers on Indigenous older adults among Black, Hispanic, and White peers. It examines whether structural inequities (educational attainment, low incomes) and discriminatory experiences (everyday discrimination, major lifetime discrimination) are associated with racial inequities in cognitive health. Using 14 years (2006-2020) of restricted Health and Retirement Study (N = 27,327) data, we estimated cognitive trajectories among Indigenous, Black, Hispanic, and White older adults using mixed effect growth curve models. Models were adjusted for discrimination experiences and relevant covariates. We found that everyday discrimination experiences were negatively associated with cognition at baseline and over time. Major lifetime discrimination experiences were associated with lower baseline cognition scores and positively with cognitive change over time. Indigenous older adults who experienced high levels of everyday discrimination had significantly lower cognition scores at baseline than any other race or ethnicity. We also found that Indigenous older adults primarily attribute perceived discrimination experiences to their age, ancestry, and race. Indigenous older adults have a unique profile that places them at elevated risk for cognitive pathology in later life, and structural inequities are powerfully associated with many of these risk factors. Addressing these inequities will require the development of policies, programs, and research agendas that target the fundamental causes of these discriminatory stresses: structural inequities in education and socioeconomic opportunities.

